# Using Self-Organizing Neural Network Map Combined with Ward's Clustering Algorithm for Visualization of Students' Cognitive Structural Models about Aliveness Concept

**DOI:** 10.1155/2016/2476256

**Published:** 2015-12-27

**Authors:** Nurettin Yorek, Ilker Ugulu, Halil Aydin

**Affiliations:** Faculty of Education, Dokuz Eylul University, Buca, 35150 Izmir, Turkey

## Abstract

We propose an approach to clustering and visualization of students' cognitive structural models. We use the self-organizing map (SOM) combined with Ward's clustering to conduct cluster analysis. In the study carried out on 100 subjects, a conceptual understanding test consisting of open-ended questions was used as a data collection tool. The results of analyses indicated that students constructed the aliveness concept by associating it predominantly with human. Motion appeared as the most frequently associated term with the aliveness concept. The results suggest that the aliveness concept has been constructed using anthropocentric and animistic cognitive structures. In the next step, we used the data obtained from the conceptual understanding test for training the SOM. Consequently, we propose a visualization method about cognitive structure of the aliveness concept.

## 1. Introduction

Although biology is named as “a science of the living things,” a clear definition of “aliveness” concept cannot be made. Because of scientific uncertainty as well as moral, legal, and theological aspects in this concept, it is difficult to make an exact definition [1]. Therefore, aliveness emerges as one of the most difficult concepts to be explained. Perhaps because of this difficulty, it is seen that there are relatively small numbers of studies examining the concept of aliveness as a main theme in the literature. Studies are generally focused on the classification of living things and mainly animals by the students (examples from other researches on the subject are given below). The studies in the field of cognitive psychology and neuroscience were mainly focused on the studies consisting of category-specific knowledge deficit or category-specific impairment about the living things (e.g., [2–8]) and the mental representation of living/nonliving distinction with the examination of the brain with modern imaging methods [9–11].

Bardel [12] revealed some conceptual models about the aliveness concept. Two of them are listed as animistic and vitalist models. In animistic model, the concepts of aliveness and motion are discussed. It is explained that motion is not only intrinsic to the living things by looking at the fact that vehicles such as cars and planes move. Also according to this model, it was stated that animals would be seen as in the forefront in terms of aliveness compared to plants, and as a result of this, it was emphasized that “animistic misconceptions” would occur. In addition to this definition of Bardel [12], it was seen that there were many studies containing the aliveness identified with motion especially in early childhood (e.g., [13–18]). Besides, there are some studies indicating the existence of animistic thinking about living things also in many elderly people [19]. Also in studies carried out in different age groups, it was emphasized that motion lays behind the fact that the subjects were interested in animals rather than plants [20, 21]. Babai et al. [22] argued that aliveness was entirely associated with motion (movement = alive) as a naive and intuitive concept, and this was riveted by protecting its existence until adulthood.

Dellantonio et al. [23] suggested that categorization as an animate/inanimate was more fundamental according to distinction such as living/nonliving or biological/nonbiological. According to this distinction, concept of motion was associated with aliveness. In the same study, it was argued that human was in a different position in terms of “vitality” and was shown in a separate category. As a result, it was stated that the perception of aliveness followed up a row in the form of “human-animal-plant” [24].

In their research about aliveness, Caravita and Falchetti [25] addressed the question “Is a piece of bone taken from a live body also alive outside the body?” to the subjects. Most of the students answered this question as “yes,” and they mostly showed the style marks of motion, structure, function, and the bone as evidence. As a result of an interesting research focused on the genetic basis of living/nonliving distinction, it was reported that aliveness concept was an innate category organized in semantic memory [26].

In his researches examining the classification of animals by the students, Braund [27, 28] expressed that the alternative concepts, misconceptions, naive theories, and some preconcepts developed against scientific concepts by the students were clear barriers against the teaching of scientific subjects. In his researches stating that the students were mostly interested in vertebrates, Braund [27, 28] revealed that 12-year-olds perceived the turtle like a slug and grouped it as an invertebrate animal. Some of the 12- and 15-year-old students described the penguin as bird, some of them described it as fish, and some of them described it as mammal. This situation showed that students took into account the external appearances of the living things while reconstructing the aliveness concept as well as motion [16].

In his study investigating the “personal taxonomic criteria” used by the students while classifying animals, Kattmann [29] suggested that students mostly used nonscientific classification criteria (swimmers, four-legged, two-legged, flying, etc.). Also, he stated that nontaxonomic criteria were at higher rate than the taxonomic criteria used. Kattmann [29] stated that students mostly took into account the external appearances rather than biological classification while classifying animals, and they performed grouping according to the motion forms (flying, swimming, and so on) that they used for the purposes of the place they lived in and change of location. Moreover, he stated that this method used by the students to classify the animals remained unchanged even though they were taught biological classification. Similar results were reported also in the studies carried out on Turkish students [30].

Some researches examining the thoughts of the students about plants are found in the literature even though they are few in number. In one of these, it was stated that students mostly evaluated the plants by their anatomic properties and external appearances while grouping them (Tunnicliffe & Reis, 2000). Wandersee and Schussler [31, 32], as botanist and biology educators, investigated the reasons why people showed interest in animals rather than plants. As a result of their studies, they formulated the plant blindness theory as a new concept. Wandersee and Schussler [31, 32] described this theory as follows: incomprehension of the value that plants had for the atmosphere and the human life, inability to appreciate the aesthetic and biological properties of the plant kingdom, and, consequently, coming to the conclusion that animals were more valuable than plants in terms of benefit to humans with an anthropocentric perspective.

There are numerous studies related to artificial intelligence approach in which conceptual modeling is “*generally*” evaluated (some of the leading ones, [33–36]). Besides, there are also studies about cognitive structuring modeling of concepts in relation to living creatures. Among these, we see that studies in which texts that include animal names (mostly simple sentences) are analyzed by using SOM are much more common [37, 38]. Furthermore, Ritter and Kohonen [37] have formulated the* self-organizing symbol map* by using logical similarities of some animals (duck, dog, cat, lion, etc.) and their characteristics (being small, being big, having 2 legs, having 4 legs, liking to fly, liking to swim, etc.).

Although it is suggested that anthropocentric and animistic misconceptions are effective in cognitive structuring of aliveness concept, it is not that easy to explicitly visualize and expose that structuring. Yorek et al. [24] have questioned whether there can be a distinction among living creatures due to aliveness based on “animistic-anthropocentric model” they have developed. In another study, which uses the same model, subject was evaluated with fuzzy and rough set approaches and a mathematical model was suggested [39]. We initiated this study with the question “How can we use SOMs in analyzing and visualizing cognitive structure of aliveness concept?” with the motivation of the idea of so called mathematical approach. How we gathered data and how we realized the analysis are explained in the following chapters of this study.

## 2. Artificial Neural Networks

Artificial neural networks (ANNs) are mathematical models inspired by biological neural networks contained in human brain. Having similar characteristics to those of biological neural networks (i.e., consistency, flexibility, parallel function, tolerance to errors, etc.), these systems attempt to learn tasks and determine how they will react to new tasks by means of creating their own experiences through the data obtained by using the predetermined samples [40].

**Figure 1:**
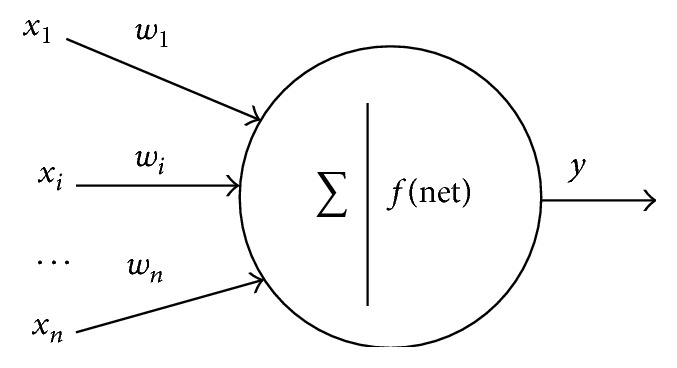
Representation of an artificial neuron.

Neural networks can be used to model complex relationship without using simplifying assumptions, which are commonly used in linear approaches [41]. The other advantages of the ANNs are the ability to represent both linear and nonlinear relationships, the ability to learn these relationships directly from the data used, not needing to take into account detailed information of structures and interactions in the systems, and the fact that they are regarded as ultimate black-box models. At least in some cases if not always, that is, for prediction using the trained network, the ANN systems are alternative to experimentation and save a lot of time which may have been consumed since experimentation is so difficult and in some cases is impossible. Artificial neurons based on biological neurons were first defined by McCulloch and Pitts [42]. McCulloch-Pitts (MCP) neuron model is given in Figure 1.

In all neural network models, *x*_*i*_ input values are multiplied by *w*_*i*_ connection weights and then summed up. Summation unit is compatible with the body of biological neuron. It sums up weighted inputs and then gives the net output (*y* = *f*(net)).

### 2.1. Self-Organizing Maps

A self-organizing map (SOM, also known as Kohonen map) is a type of the artificial neural algorithm and is based on unsupervised learning. The structure of SOMs is composed of two layers fully attached to each other: input layer and Kohonen layer [43]. Kohonen layer is also the layer where the map is formed that will ensure the observation of clustering in the data set. Hidden layers are not in question as in prediction or classification studies. Neurons in the Kohonen layer are generally arranged two-dimensionally. The number of neurons in the input layer is equal to the number of variables used. Each neuron in the input layer is connected to each neuron in Kohonen layer as feed forward. Inputs can be calculated by the following for the Kohonen layer:

(1) $$\begin{array}{c} y_{j}=\overset{d}{\underset{i=1}{\sum }}w_{ji}x_{i}. \end{array}$$

*w*_*ji*_ in (1) is the weight of the connection outgoing from the input neuron represented by *i* to the neuron represented by *j* in Kohonen layer. The weight vectors are collectively known as the SOM's memory [44]. *d* represents the number of variables. When considering the winner-take-all paradigm, the neuron taking the highest *y*_*j*_ value in Kohonen layer will be the winner neuron.

The SOM algorithm firstly assigns small random values to the connections between input layer and the Kohonen layer. Then, the algorithm undergoes three essential processes. These are competition, cooperation, and adaptation [45].

*Competition Process*. A random observation (student) is selected from the data set. This observation can be expressed as *x* = (*x*_1_, *x*_2_,…, *x*_*d*_). In Kohonen layer, the expression of weights of the neuron *j* is possible as follows:

(2) $$\begin{array}{c} w_{j}=\left(\;w_{j1},w_{j2},\ldots ,w_{jd}\;\right),\;j=1,2,\ldots ,d. \end{array}$$

The expression of *d* in (2) represents the total number of neurons in the Kohonen layer. In order to find the best match of weight vectors *w*_*j*1_, *w*_*j*2_,…, *w*_*jd*_ with *x* input observation, *w*_1_ *x*, *w*_2_ *x*,…, *w*_*d*_ *x* scalar products are calculated and the largest one is selected. The criteria of finding the best match based on the selection of the largest one of the scalar products are equal to the mathematical maximization of Euclid distance between *w*_*j*_ vector and *x*. Therefore, the index of the winning neurons for observation is calculated as follows:

(3) $$\begin{array}{c} i\left(\;x\;\right)=\underset{1\leq j\leq d}{\arg\;\min}\left\|\;x-w_{j}\;\right\|. \end{array}$$

For each input in the competition process, neurons in the model are in competition with each other.

*Cooperation Process*. In the cooperation process, a topological neighborhood is determined, and the cooperating neurons will settle according to the topological neighborhood such that the winner neuron will be at the center. The winner neuron determines the topological value of the neurons affected by competition; therefore, cooperation is ensured between nodes.

*Adaptation (Synaptic Compatibility)*. Neurons affected by competition arrange their synaptic weights according to the example. The new weight vector in (*s* + 1) cycle of neuron *j* is calculated as follows:

(4) $$\begin{array}{c} w_{j}^{\left(s+1\right)}=w_{j}^{s}+\eta \left(\;s\;\right)h_{j,i\left(x\right)}\left(\;s\;\right)\left(\;x-w_{j}^{\left(s\right)}\;\right). \end{array}$$

Here, *η*(*s*) is the learning parameter and *h*_*j*,*i*(*x*)_(*s*) is the neighborhood function.

**Figure 2:**
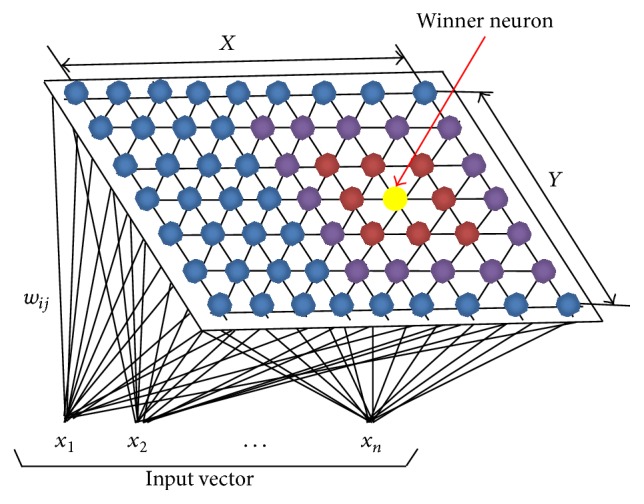
SOM input and output layers.

Neurons are connected to each other by neighborhood relation. This neighborhood relation determines the structure or topology of the map.  Figure 2 represents a simple SOM. Kohonen layer consisting of 9 × 7 neurons appears in the figure. The input vector is represented by *x* and there are variables in this vector (it is expressed that *n* number of variables exist in the expression of *x*_*n*_). The weight vector is represented by *w* and it contains the weights outgoing from each variable to the each neuron. The yellow-colored neuron represents the winner neuron and the surrounding neurons are its neighbors.

### 2.2. The Ward Clustering in General

The Ward classic clustering method is a hierarchical agglomerative cluster algorithm. Clustering process is initiated by accepting each node as a separate cluster. Then, at each stage of the algorithm, the clusters with minimum distances between themselves (according to the distance measure defined by a specific algorithm) are combined in pairs. This smallest distance is called the Ward distance and defined as follows:

(5) $$\begin{array}{c} d_{rs}≔\frac{n_{r}\cdot n_{s}}{n_{r}+n_{s}}\cdot {\left\|\;{\overset{-}{x}}_{r}-{\overset{-}{x}}_{s}\;\right\|}^{2}. \end{array}$$

Here, *r* and *s* represent the two distinct clusters, *n*_*r*_ and *n*_*s*_ represent the data points of two clusters, *x*_*r*_ and *x*_*s*_ represent the center of gravity of the clusters, and ‖·‖ is Euclidean norm.

Starting from the full distance matrix (lower triangle matrix as the distance measure is commutative), a row and a column are removed in each step until the matrix is completely cleared and only one cluster will remain (and a different row and column are updated).

The mean and cardinality of the new cluster built as a product of the combination phase are calculated as follows:

(6) x−rnew≔1nr+ns·nr·x−r+ns·x−s,nrnew≔nr+ns.

### 2.3. The SOM-Ward Clustering

The two main ways to cluster data are hierarchical and partitive approaches. The hierarchical methods can be further divided into agglomerative and divisive algorithms, corresponding to bottom-up and top-down strategies, to build a hierarchical clustering tree. Of these, agglomerative algorithms are more commonly used than the divisive methods [46].

The SOM-Ward clustering approach is a two-level clustering approach that uses Ward's clustering algorithm to determine the SOM and clustering results. The Ward clustering algorithm is an agglomerative hierarchical clustering method [18, 47]. Agglomerative clustering algorithms usually have the following steps [46]:

1. Initialize: assign each vector to its own cluster.
2. Compute distances between all clusters.
3. Merge the two clusters that are the closest to each other.
4. Return to step (2) until there is only one cluster left.

In the SOM-Ward clustering approach, process begins by accepting each node as a separate cluster. Until one cluster will remain on the map, clusters with minimum Euclidean distance between themselves are combined with each other in pairs. While determining the distance, both the Ward distance and the topological properties of SOM are taken into account. In other words, the distance between two nonassociated clusters is considered as infinite and only the associated clusters are combined. Low SOM-Ward distance value represents a more natural clustering for the map, and high value represents an artificial clustering for the map. By this means, users can select the optimal cluster number in a flexible manner.

### 2.4. Cognitive Structural Modeling and SOMs

Although using of SOM is recommended in conceptual modeling studies [34, 35], it is suggested that SOM modeling in executive functions of brain such as reasoning and language faces some difficulties. Since self-organized maps reflect simple distance relations among input vectors, they mostly characterize lower levels of perception. For high level processing, discrete symbols are needed. Maps formed by these symbols in brain are composed of logically related symbols coating neighboring areas [37]. Similarly, Gärdenfors [33, 48], while explaining representing information on conceptual level in his* conceptual spaces* theory, associates quality dimensions with* similarity* and* distance* concepts [34, 49, 50].

When the neural adaptation law (4) is applied to a vector variables set, a topographic map is being obtained that shows logical distance among symbols. But logical relatedness between different symbols cannot be directly determined by their encodings. At least in learning process, symbols should be regularly presented due to their attribute values. From these, we can come up with the conclusion that symbols with similar characters are represented close to each other on map [37].

Results obtained from text analysis prove that SOMs can be safely used in high dimensional data analysis such as independent component analysis (ICA), principal component analysis (PCA), and singular value decomposition (SVD) [35, 38, 51]. In these studies, SOMs can be used single, while they can also be combined with different models (Bayesian, etc.) [36].

This study reports on the discussion of how to utilize the SOMs in the modeling and visualization of the cognitive construction of aliveness concept. In this regard, the questions that we searched for an answer to are as follows: Which living things are primarily associated with the aliveness concept by the subjects? and How can we use SOMs in modeling and visualization of cognitive construction of aliveness concept?

## 3. Method

Qualitative data obtained from student answers to open-ended questions were used to create SOM-Ward model.

### 3.1. Subjects

The participants included 100 students (55 female and 45 male) who were selected via cluster sampling method from nine high schools in Izmir, a large city in western Turkey. Schools accepted students from different parts of the city and students varied in terms of socioeconomic status.

### 3.2. Data Collection

In this study, a conceptual understanding test was used. The test included open-ended questions and was developed by researchers. In addition, to clarify vague concepts and to obtain in-depth information about the topics, interviews were conducted with students and teachers. The final version of the test used in this study is presented in Table 1.

## 4. Results

### 4.1. Conceptual Understanding Test Results

**Question 1 .**

When analyzing all of 10 living things written by the students, those most frequently repeated were, respectively, human, dog, cat, mouse, and rabbit. It was observed that 107 (74.31%) of 144 different living things written were animals, 26 (18.05%) of them were plants, and the remaining 11 (7.64%) were other living things.

Those that were most frequently written in animals (51.43%) were the mammals. It was observed that it was the human which was mostly written to the first rank among 10 living things. When looking at the answers of the students who wrote at least one plant name among the answers, it was observed that they wrote plant to the 6th rank among 10 living things.

According to this, it could be said that students structured the “aliveness concept” by associating with the animals, particularly with human. In this structuring, it was believed that plants and other living things came after human and animals in terms of aliveness.

**Question 2 .**

When analyzing the answers of the students to the second question about the separation of living things (types of living things) into certain groups, about one-third of the students (35%) created the groups consisting of “animal, plant, and human.” The most interesting result of the answers given to this question was that of 24% of students; in other words, one out of every four students created just “animal” groups during grouping.

**Question 3 .**

When analyzing the answers of the students to the third question about the position of human within all living things, 78% of students emphasized the human's ability of being intelligent and of thinking. Starting from this point, they defined the position of human as the “most excellent,” “most sophisticated,” and thus “the most supreme being.”

### 4.2. Creating the SOM-Ward Model

#### 4.2.1. Data Preprocessing for Training of the SOM-Ward Model

For the training of the SOM-Ward model, the answers given to the first question were taken into account by the students' conceptual understanding test. The data obtained from the other questions were used in the verification of the answers given to the first question and in the interpretation of the SOM-Ward model.

Firstly, 144 living things written by the students were collected under 10 groups (Table 2). While these groups were determined, animals were mostly included as students wrote the name of animal at the most. Then, a code was defined for each group (Table 3).

In the next step, living things written by each student (S_1_, S_2_,…, S_100_) were coded and tabulated by preserving their ranks (R_1_, R_2_,…, R_10_). An example of students' responses is seen in Tables 4 and 5, respectively.

In the next phase, standardization process was performed because the data set presented in Table 5 is categorical. However, there was no difference among these values as these values (the numbers from 1 to 10) symbolized the groups. In case of using these data as such, analysis results will be incorrect as “1” will bear the meaning of “the lowest” and “10” will bear the meaning of “the highest.”

In this respect, a new arrangement was made for figures to denote the same meaning for the variables. A new table was created by calculating on which ranks the students wrote the living things on average. In this case, the living thing with low average rank would be more important in terms of “aliveness” compared to the living thing with high average rank. And this value is of the same meaning for each living thing group. We determined the average rank of the living things. The average rank was determined as follows: for a group of living things we found, from one to 10, at which ranks (R_1_, R_2_,…, R_10_) they were listed. Then, for each line, these values were added together and then divided by the total frequency, for example, code 9 for the student S_1_ in Table 5; this code was listed five times on the 3rd, 5th, 6th, 9th, and 10th ranks. Accordingly, the average rank was calculated as (3 + 5 + 6 + 9 + 10)/5 = 6.60. The following is an example which was formed according to Table 5 in this way (Table 6).

The data in Table 6 are still not suitable for training the SOM. Because if “human” is mentioned as the first object, that is, as the most prominent example of “living,” “human” gets a value of 1 according to the rank in the list (plus an averaging over objects belonging to the same biological category, which is fine). However, this leads to the emergence of a problem. If any entity belonging to biological category “fish” is not mentioned at all by a student in the 10 first alive objects list, it gets a value 0.00 (see, e.g., first row of Table 6). This means that, in this metric of similarity, “no mention” is the closest to “mentioned as the first thing in the list.” Of course, “no mention” should be most similar to “mentioned as the last thing in the list.” If something is not mentioned by a subject, it is not a very prototypical sample of “alive” and therefore should have a low value of “aliveness.”

All the data in Table 6 were subtracted from 11 for eliminating the problems; that is, if “human” is mentioned as first thing in the list of 10 objects, it gets “aliveness” value of 11 − 1 = 10. If it is the second, it gets value of 9. And so on, the 10th object gets value of 1. Then, something that is not mentioned at all, such as the “fish” for student S_46_, can then get value of 0. Accordingly, Table 7 was created.

The values exemplified in Table 7 were calculated for each student. This 10-dim data (*x*_1_, *x*_2_,…, *x*_10_) were used in the training of the SOM-Ward model (Table 9).

### 4.3. The SOM-Ward Model Results

For clustering and visualization of cognitive construction of aliveness concept, the SOM-Ward model was trained by batch training algorithm. Matlab and Viscovery SOMine software were used in the creation of SOM [52].

#### 4.3.1. Clustering and Visualization of the Clusters

The SOM cannot fully give detailed information about the clusters. In the Viscovery SOMine program, hierarchical clustering algorithm and the SOM are used together, and the interpretation of the resulting maps will be easier. This clustering algorithm operates as follows: firstly, each student (observation) is divided into a separate cluster. In this way, the number of clusters will be equal to the number of observations. Then, in each step, observations which are the closest to each other are combined according to the SOM-Ward distance measure. In this special distance unit, both the distance of the two clusters and the location on the map are taken into account. All 10 variables were used to reveal the clusters in the data set.

**Figure 3:**
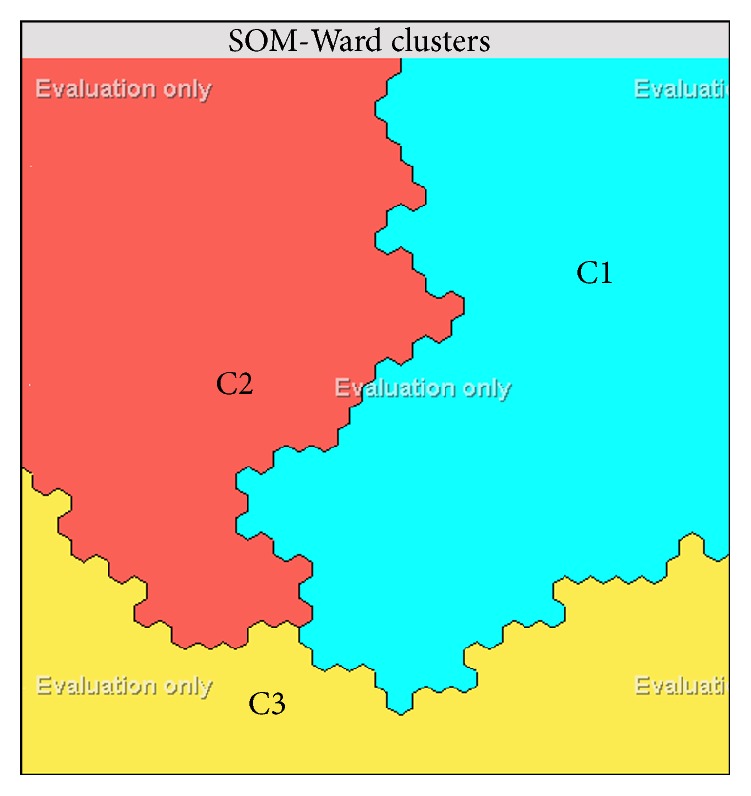
SOM-Ward clusters after training.

To what extent will the neurons behave competitively with one another in the training phase is determined by the tension parameter of the map. The smaller this value is, the more the map tries to harmonize itself to the data set. This value varies between 0.3 and 2 and it was determined as 0.5 in this study. 1000 neurons were used in Kohonen layer and the emerging clusters are shown in Figure 3.

**Figure 4:**
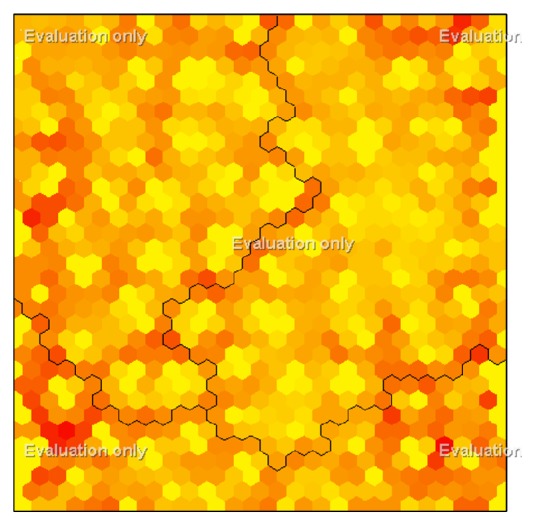
U-matrix.

U-matrix emerged after the formation of Ward agglomerative hierarchical clustering and SOM together is shown in Figure 4. Number of clusters will be determined with the help of this matrix. Light colors in this map represented that there were more students; therefore, there was a clustering. Dark colors in this map showed that there was not an observation in that part of the map. Therefore, dark colors on the map determined the cluster boundaries.

The number of clusters can be determined by looking at U-matrix; however, there may be some personal judgments as people's perceptions will come to the forefront. In the literature, different methods in determining the number of clusters are recommended using U-matrix [53, 54].

Depending on our training data (Table 7), Kohonen layer is divided into three clusters. Related to these clusters, observation percentage in clusters and the percentage of each variable present in the related cluster are given in Table 8.

Generally, in clustering studies, it is seen that each cluster is named. Such a naming study is usually performed by the researcher. Clusters were named also in this study.

Distinguishing characteristics of clusters can be expressed as follows.

*C1-Anthropocentric Cognitive Structure*. 39% of students in the data set taken into account in this study are in this segment. It is seen that human, mammals, and plants stand out. 

*C2-Primary Animistic Cognitive Structure*. 32% of the data sets are in this segment. Domestic and/or familiar animals stand out, for example, fish, birds, and domestic mammals. 

*C3-Secondary Animistic Cognitive Structure*. 29% of students are clustered in this segment. Wild and/or unfamiliar animals, for example, wild mammals, reptiles, and invertebrates, prokaryotes, protists, and funguses stand out.

**Figure 5:**
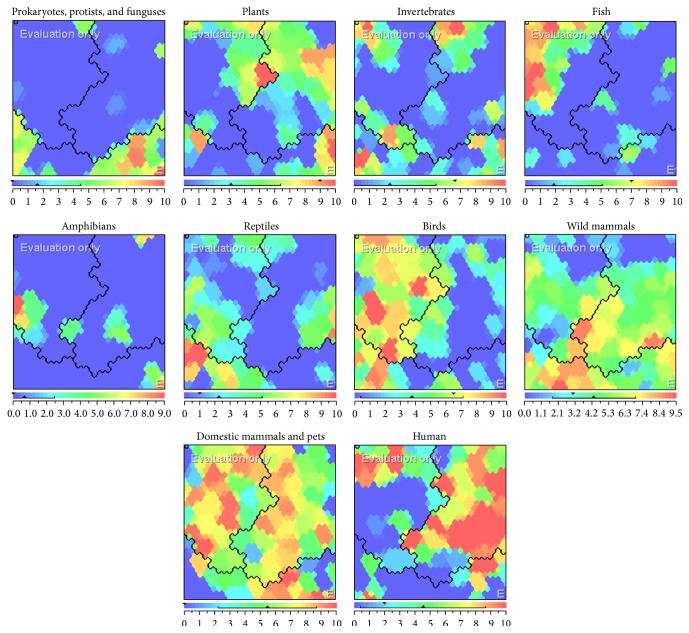
The component planes of the map.

It is highly apparent that the spatial order of the responses has captured the essential “family relationships” among the living things [37]. Clusters responding to, for example, “human,” occupy the right part of the lattice, clusters responding to “familiar animals” such as “birds,” “domestic mammals,” and “pets” gather towards the left, and clusters responding to more “wild” species such as “reptiles,” “wild mammals,” and “invertebrates” aggregate in the lower middle. Within each cluster, a further grouping according to similarity is discernible [37]. The component planes of the map are shown in Figure 5.

The resulting SOM-Ward model consists of three clusters. The component planes (Figure 5) shows the distributions of each variable (groups of the living things) across the map, on which the color scale visualizes the distribution of each variable over different clusters. Warm colors indicate more significant for construction of aliveness concept, while cold ones indicate the reverse; for example, fishes were mainly found in cluster C2 and human in C1.

## 5. Conclusion

Cluster analysis is a dimension reduction method. In this study, cluster analysis was applied to the data set obtained from the students with regard to the aliveness concept with the help of SOM. 10 variables of 100 students were expressed with the help of two-dimensional maps. As a result, 3 clusters which we called C1, C2, and C3 emerged with regard to the cognitive construction of aliveness concept. Properties of these clusters and the emergence of different colors according to the input variables are thought to be an important step for us to “be able to understand” the aliveness concept.

According to our literature survey, it was observed that the cognitive construction of aliveness concept was closely associated with anthropocentrism and animism [13–19, 22, 24, 31, 32, 39, 55]. The results obtained from this study appeared to be consistent with the literature. As an additional property, the visualization of the results by using SOM enabled the clusters to form the cognitive structure and the characteristics of this cluster to be seen clearly.

Another issue is the optimization of the structures of the SOMs. For instance, different structures can be obtained by changing the number of neurons in the output level. Similarly, different maps will emerge when learning parameter is changed. Another issue is the fact that the results of the analysis will actually show deviation when irrelevant variables are used. Different methods have been developed to optimize the structures of the SOMs.

This classification process performed with the help of SOMs can be renewed by a different data set. For instance, there might be some variables that might increase the performance of the clustering analysis when they are removed from the analysis. The determination and removal of such variables with other methods may ensure analysis to have higher performance. For this, primarily, determining a reliable performance criterion is required.

Recent analysis that became possible by increasing the processing capacity of the computers can be applied for modeling in the field of cognition and neuroscience. It can be expected that the SOMs can also be used to solve different problems in this field due to the power of visualization they have.

## Figures and Tables

**Table 1:** Conceptual understanding test.

Questions		Aim
1	Write the names of the first 10 living things coming to your mind.	Uncovering which living thing is primarily associated with the aliveness concept.

2	It is estimated that there are millions of species living on Earth. If you were asked to classify all the living things (types, species) into main groups, without leaving anyone out, at least how many groups could you form?	Uncovering which properties of the living things are paid attentions by the students while dividing them into groups, how they created their own taxonomic groups, how they expressed this conceptually, or how they called the groups.

3	When considering the all living things, what is the place (position) of human? Please explain.	Clarifying the reasons why the students evaluated the human in a separate category while grouping the living things in the nature based on their own statements.

**Table 2:** Grouping of living things written by the students (as it is written).

1	2		3		4	5
Euglena	Daisy	Blackberry	Parasite(s)	Sea cucumber	Fish(es)	Frog
Bacterium	Plant(s)	Lotus	Earthworm	Tick	Shark	
Amoeba	Tree(s)	Jasmine	Spider	Snail	Stingray	
Mushroom	Rose	Mistletoe	Beetle(s)	Hydra		
Algae	Flower(s)	Purslane	Butterfly	Invertebrate		
Paramecium	Cactus	Daffodil	Bee	Jellyfish		
Monera	Grass	Kiwi	Grasshopper	Calamari		
Protists	Fern	Snowdrop	Scorpion	Mussel		
Plasmodium	Tulip	Willow	Fly	Mantis		
Plankton	Chrysanthemum	Geranium	Ant	Sponge		
Planaria	Sunflower	Begonia	Caterpillar	Cockroach		
	Hyacinth	Violet	Cancer	Ladybug		
			Millipede	Octopus		

6	7	8		9	10	

Snake	Bird(s)	Lion	Squirrel	Dog	Human, human names	
Crocodile	Eagle	Bear	Panda	Cat		
Lizard	Bat	Tiger	Monk seal	Mouse		
Turtle	Penguin	Monkey	Leopard	Rabbit		
Reptile	Parrot	Giraffe	Weasel	Cow		
Chameleon	Falcon	Elephant	Panther	Horse		
Iguana	Pigeon	Whale	Vertebrate	Donkey		
Dragon	Duck	Wolf	Tapir	Sheep		
	Cock, Rooster	Fox	Gazelle	Ox		
	Chicken, Chick	Gorilla	Cougar	Camel		
	Owl	Dolphin	Skunk	Goat		
	Partridge	Kangaroo	Cheetah	Mammal(s)		
	Sparrow	Koala	Rhinoceros	Calf		
	Hawk	Hippopotamus	Otter	Lamb		
	Canary	Zebra	Anteater	Buffalo		
	Ostrich	Hedgehog	Hyena	Lama		
	Vulture	Animal(s)	Pork	Mule		
			Lynx	Bull		

**Table 3:** Groups' definitions (according to biological classification).

Code	Groups	Explanation
1	Prokaryotes, protists, and funguses	Bacterium, algae, amoeba, unicellular eukaryotes, Mushrooms, and so forth
2	Plants	Flowering and nonflowering plants
3	Animals	All invertebrates, insects, mollusks, and so forth
4	Animals	Fishes
5	Animals	Amphibians
6	Animals	Reptiles
7	Animals	Birds
8	Animals	Wild mammals
9	Animals	Domestic mammals and pets
10	Animals	Human

**Table 4:** An example of students' responses (first question of conceptual understanding test).

Students	Living things written by the students									
	R_1_	R_2_	R_3_	R_4_	R_5_	R_6_	R_7_	R_8_	R_9_	R_10_
S_46_	Human	Monkey	Donkey	Bird	Cow	Camel	Frog	Turtle	Mouse	Cat
S_47_	Cat	Dog	Tree	Eagle	Dolphin	Pigeon	Lion	Whale	Daisy	Snowdrop
S_48_	Lion	Human	Plant	Panda	Snake	Dolphin	Zebra	Penguin	Bear	Wolf
S_49_	Human	Plant	Dog	Algae	Bacterium	Mushroom	Bird	Lion	Leopard	Cat
S_50_	Dolphin	Dog	Human	Daisy	Rose	Ox	Bear	Whale	Monkey	Chrysanthemum

**Table 5:** Coding of students' responses.

Students	Coded data of living things									
	R_1_	R_2_	R_3_	R_4_	R_5_	R_6_	R_7_	R_8_	R_9_	R_10_
S_46_	10	8	9	7	9	9	5	6	9	9
S_47_	9	9	2	7	8	7	8	8	2	2
S_48_	8	10	2	8	6	8	8	7	8	8
S_49_	10	2	9	1	1	1	7	8	8	9
S_50_	8	9	10	2	2	9	8	8	8	2

**Table 6:** A sample of average ranking of the living things.

Students	Monera, protists, and funguses	Plants	Invertebrates	Fishes	Amphibians	Reptiles	Birds	Wild mammals	Domestic mammals	Human
	*x*_1_	*x*_2_	*x*_3_	*x*_4_	*x*_5_	*x*_6_	*x*_7_	*x*_8_	*x*_9_	*x*_10_
S_46_	0.00	0.00	0.00	0.00	7.00	8.00	4.00	2.00	6.60	1.00
S_47_	0.00	7.33	0.00	0.00	0.00	0.00	5.00	6.67	1.50	0.00
S_48_	0.00	3.00	0.00	0.00	0.00	5.00	8.00	6.17	0.00	2.00
S_49_	5.00	2.00	0.00	0.00	0.00	0.00	7.00	8.50	6.50	1.00
S_50_	0.00	6.33	0.00	0.00	0.00	0.00	0.00	6.25	4.00	3.00

**Table 7:** Inputs for training the SOM-Ward Model.

Students	Monera, protists, and funguses	Plants	Invertebrates	Fishes	Amphibians	Reptiles	Birds	Wild mammals	Domestic mammals	Human
	*x*_1_	*x*_2_	*x*_3_	*x*_4_	*x*_5_	*x*_6_	*x*_7_	*x*_8_	*x*_9_	*x*_10_
S_46_	0.00	0.00	0.00	0.00	4.00	3.00	7.00	9.00	5.60	10.00
S_47_	0.00	4.33	0.00	0.00	0.00	0.00	6.00	5.67	9.50	0.00
S_48_	0.00	3.00	0.00	0.00	0.00	6.00	3.00	5.17	0.00	9.00
S_49_	6.00	9.00	0.00	0.00	0.00	0.00	4.00	3.50	5.50	10.00
S_50_	0.00	5.33	0.00	0.00	0.00	0.00	0.00	5.25	7.00	8.00

**Table 8:** Clusters and descriptive statistics.

Groups of the living things	Clusters		
	C1	C2	C3
	39%	32%	29%
Prokaryotes, protists, and funguses	0.41	0.20	4.81
Plants	3.59	2.81	2.75
Invertebrates	1.81	2.43	3.06
Fishes	0.54	4.78	0.48
Amphibians	0.49	0.84	0.62
Reptiles	1.62	2.32	3.08
Birds	1.73	6.50	3.50
Wild mammals	4.12	3.36	5.62
Domestic mammals and pets	5.76	6.33	4.06
Human	7.15	3.30	2.34

**Table 9:** Inputs data for training the SOM-Ward Model.

Students	Monera, protists, and funguses	Plants	Invertebrates	Fishes	Amphibians	Reptiles	Birds	Wild mammals	Domestic mammals	Human
	*x*_1_	*x*_2_	*x*_3_	*x*_4_	*x*_5_	*x*_6_	*x*_7_	*x*_8_	*x*_9_	*x*_10_
S_1_	0	0	0	0	0	3	8	4.67	9.5	5
S_2_	0	3.5	0	0	0	0	0	3.5	9	7
S_3_	0	0	0	0	0	6.33	9	7	4	0
S_4_	0	0	2.5	9	0	0	3	5.6	0	10
S_5_	0	0	0	0	0	0	0	0	0	5.5
S_6_	6	5	3	0	0	0	0	1.5	8.5	4
S_7_	0	0	3	0	0	0	0	5.25	6.2	0
S_8_	9.5	0	0	0	0	0	2	3.75	5	8
S_9_	0	0	0	0	0	5.33	8	1.5	7	0
S_10_	0	0	0	0	0	4.5	0	8	2.5	0
S_11_	0	0	0	0	0	0	1	2	0	6.5
S_12_	0	2	3	10	0	4	9	1	6.67	6
S_13_	0	0	0	1	0	0	0	4.5	7.2	0
S_14_	0	0	0	9	4	3	10	4.33	7.5	0
S_15_	0	0	8	9	0	0	6	3	7	10
S_16_	0	5	8	9	0	2	7	0	0	10
S_17_	0	4.33	6	5	0	0	0	0	0	7
S_18_	0	0	5.33	0	0	4	0	8.5	4.5	0
S_19_	0	6	5	0	0	0	0	3.75	7.5	7
S_20_	3.5	0	5.5	0	0	0	8	4.5	0	10
S_21_	0	0	0	0	0	0	3	5.6	7.67	1
S_22_	0	4	0	0	0	2	0	4.75	6.67	10
S_23_	0	0	1.5	0	0	0	4	7	6.33	8
S_24_	1	10	9	0	0	0	2	7.5	5	3
S_25_	0	8	0	5	0	0	2.5	6	2.5	10
S_26_	7	1.5	10	3	0	8	4	6	0	0
S_27_	0	0	0	0	0	3	0	4.67	6.25	10
S_28_	7	0	0	0	3	4	8	2	1	0
S_29_	5.5	3	0	0	0	10	9	8	0	0
S_30_	0	4	0	3	0	0	0	7	8	1.5
S_31_	6	7	3	0	0	0	0	7	0	10
S_32_	0	0	7	0	0	0	9.5	8	0	4
S_33_	6	4.5	1	0	0	9.5	0	3	4.5	8
S_34_	0	5.5	0	0	0	0	7	0	5.29	0
S_35_	0	5.5	0	7	0	0	8	3	6.5	10
S_36_	0	0	0	0	0	0	0	0	5	5.56
S_37_	0	6	8	0	0	3	4	2	6.67	0
S_38_	0	1.5	0	0	0	5	0	6.33	7.67	0
S_39_	0	7	0	0	0	0	10	4	2.5	0
S_40_	1.5	0	0	0	0	5.5	0	9	4.67	9
S_41_	6.25	9	0	0	0	0	0	7	0	8
S_42_	7	5.5	3	0	0	9	5	0	0	0
S_43_	0	10	1	0	0	2	7.5	6	6	3
S_44_	0	0	9	0	0	3	10	4.5	5.5	2
S_45_	0	0	5.5	0	0	2.5	7	8	6	2
S_46_	0	0	0	0	4	3	7	9	4.4	10
S_47_	0	3.67	0	0	0	0	6	4.33	9.5	0
S_48_	0	8	0	0	0	6	3	4.83	0	9
S_49_	6	9	0	0	0	0	4	2.5	4.5	10
S_50_	0	4.67	0	0	0	0	0	4.75	7	8
S_51_	0	6.5	0	0	0	0	4	7.33	4	0
S_52_	0	2	0	0	5	0	0	5.67	8.5	10
S_53_	0	0	4.6	0	7	0	1	0	5	9
S_54_	0	0	2.5	2	0	7.5	3	8.33	5	0
S_55_	0	5.33	4.33	0	0	0	6	0	5.5	9
S_56_	0	9	0	0	0	0	2.33	3	6	9
S_57_	0	9	0	0	0	1	4	4.33	6	10
S_58_	0	0	0	7	4	6	10	2.5	7.33	1
S_59_	0	0	6	2	7	1	8	4	9.5	0
S_60_	0	0	5.5	4	0	1	8	2.5	9.5	7
S_61_	0	0	1	0	0	5.5	0	6.33	4.67	10
S_62_	0	5	10	2	0	4	3	0	7.67	4
S_63_	0	0	2.5	6	0	0	8	3.33	8.67	0
S_64_	10	7	2	0	8	0	0	5	9	0
S_65_	1.5	5.75	4	0	0	0	0	0	9	7
S_66_	0	2	0	0	0	0	4	7	8	7
S_67_	0	4	4	0	2	6.5	6	0	10	0
S_68_	0	5.5	0	1	0	4	0	5.5	7	10
S_69_	0	4	0	4	0	3	7	0	9	5.5
S_70_	0	7	0	9	0	4.5	3.5	0	7	2
S_71_	0	0	0	10	0	6.5	1.5	5	9	0
S_72_	0	0	0	8	0	2	6	6.5	2	9
S_73_	0	0	2	0	0	8	3	7.4	2.5	0
S_74_	0	7	1.5	0	0	0	6	4	8.5	10
S_75_	0	4.25	0	0	0	3	6	0	8.33	4
S_76_	0	0	0	5	0	2	6	5.4	7.5	0
S_77_	0	0	2.5	9	1	0	0	7.5	6.25	0
S_78_	1	0	0	10	9	6	5	5.67	3.5	0
S_79_	0	1.5	3.5	5	0	0	7	0	7.67	10
S_80_	1	2	0	0	3	0	0	4.5	7.5	10
S_81_	4.5	1	2	0	0	6	6	9.5	0	0
S_82_	0	7	2	0	0	8	1	4.5	6	10
S_83_	0	8	0	0	0	0	0	5.5	4.67	5.67
S_84_	0	7	9	0	0	3.5	0	1.5	7.25	0
S_85_	0	0	0	0	0	0	3	7.33	1.5	5
S_86_	8.34	3	1	0	0	0	0	5	7.5	0
S_87_	9	0	8	2	0	0	0	5	1	10
S_88_	6	4	0	1	0	0	6	5	9	0
S_89_	7	5.33	6	0	2	0	3	5	10	0
S_90_	6	0	3.33	0	0	3	5	7	8.5	0
S_91_	0	7	10	8	0	2	9	3.33	5	4
S_92_	0	9	6.67	7	0	1	6.5	3	0	2
S_93_	5.6	0	3	0	0	0	6	4.5	9	0
S_94_	7	0	8	0	0	1	6	6.25	0	3
S_95_	6	9	1	0	0	10	7	2.5	8	5
S_96_	5.25	8	4	0	5	3	0	6	0	0
S_97_	6	0	0	6	0	0	0	1.5	10	3
S_98_	0	2	6.5	0	0	5	2	6	9	10
S_99_	5.5	8	0	6	0	5	0	1.5	9.5	3
S_100_	0	1.5	3	4	0	0	6.5	6.5	9	10
